# The Whole Exome Sequencing Clarifies the Genotype- Phenotype Correlations in Patients with Early-Onset Dementia

**DOI:** 10.14336/AD.2018.0208

**Published:** 2018-08-01

**Authors:** Yangqi Xu, Xiaoli Liu, Junyi Shen, Wotu Tian, Rong Fang, Binyin Li, Jianfang Ma, Li Cao, Shengdi Chen, Guanjun Li, Huidong Tang

**Affiliations:** ^1^Department of Neurology, Ruijin Hospital, School of Medicine, Shanghai Jiao Tong University, Shanghai, China; ^2^Shanghai Mental Health Center, School of Medicine, Shanghai Jiao Tong University, Shanghai, China; ^3^Department of Neurology, Shanghai Fengxian District Central Hospital, Shanghai Jiao Tong University Affiliated Sixth People’s Hospital South Campus, Shanghai, China; ^4^Department of Neurology, Ruijin Hospital North, School of Medicine, Shanghai Jiao Tong University, Shanghai, China

**Keywords:** frontotemporal dementia, Alzheimer’s disease, next-generation sequencing, variants classification

## Abstract

Our study aimed to identify the underlying causes in patients with early onset dementia by clinical and genetic exploration. We recruited a group of 38 patients with early-onset dementia. Firstly, hexanucleotide repeat expansions in *C9ORF72* gene were screened in all subjects to exclude the possibility of copy number variation. Then, the whole exome sequencing (WES) was conducted, and the data were analyzed focusing on 89 dementia-related causing and susceptible genes. The effects of identified variants were classified according to the American College of Medical Genetics and Genomics (ACMG) standards and guidelines. There were no pathogenic expansions in *C9ORF72* detected. According to the ACMG standards and guidelines, we identified five known pathogenic mutations, *PSEN1* P284L, *PSEN1*c.857-1G>A, *PSEN1* I143T, *PSEN1* G209E and *MAPT* G389R, and one novel pathogenic mutation *APP* K687N. All these mutations caused dementia with the mean onset age of 38.3 (range from 27 to 51) and rapid progression. Eleven variants with uncertain significance were also detected and needed further verification. The clinical phenotypes of dementia are heterogeneous, with both onset ages and clinical features being influenced by mutation position as well as the causative gene. WES can serve as efficient diagnostic tools for different heterogeneous dementia.

Dementia can be caused by various underlying diseases characterized by progressive deterioration of cognitive functions [1]. Early onset dementia is conventionally thought to include patients with onset before 65 years of age. In neurodegenerative diseases, early onset dementia is more likely to have a genetic cause, while late-onset dementia involves both environmental risk factors and multiple genetic loci [2, 3]. Alzheimer’s disease (AD) is the most common neurodegenerative disorder with a prevalence of 34-54% in patients with dementia. Approximately 30% of the cases of dementia before age 50 years are attributed to Alzheimer’s disease [4]. Frontotemporal dementia (FTD), the second most common cause of dementia after Alzheimer’s disease, represents 5-10% of all dementia and 10-20% cases under age 65 [5]. Both AD and FTD show high heterogeneities in clinical manifestations and genetic spectrum. The main clinical manifestations of AD are episodic memory impairment, difficulty in performing visuospatial tasks, while social behaviors are spared. However, visual, language, behavioral and dysexecutive predominant variants of Alzheimer’s disease have also been reported [6]. FTD is clinically categorized into the behavioral variant (bvFTD) and the language variant or primary progressive aphasia (PPA). There is frequent overlap between FTD and several motor diseases [7]. In some patients with early onset dementia presenting of progressive cognitive decline without memory impairment, the diagnosis is often challenging due to symptom overlap with language, behavioral and dysexecutive predominant variants of Alzheimer’s disease and FTD. Therefore, we could turn to genetic testing for a molecular diagnosis.

It is known that dormant inherited mutations in *APP*, *PSEN1*, and *PSEN2* lead to early onset Alzheimer’s disease (EOAD) and mutations in *MAPT*, *GRN* and *C9ORF72* cause familial FTD. However, mutations in these genes could only explain 13% of EOAD and 60% of familial FTD, respectively [5, 8]. Copy number variation in *C9ORF72* was a major cause of FTD in the western populations, but it was not identified in the Chinese FTD cohort [9]. Recently, the whole exome sequencing (WES) has been demonstrated to be an efficient tool for detecting novel risk factors in large samples and deliver novel insights with small numbers of patients [10-13]. However, WES in exploring the genotype-phenotype correlations in patients with dementia have been rarely reported. In this study, considering the limitation of WES to detect copy number variations (CNVs), we first screened *C9ORF72* gene and then performed WES to investigate genotype-phenotype correlations of patients with early onset dementia and clarify the clinical diagnosis of patients presenting of atypical phenotypes. The clinical effects of identified variants were classified according to the American College of Medical Genetics and Genomics (ACMG) standards and guidelines.

## MATERIALS AND METHODS

### Subjects

A total of 38 patients were included in our study. Demographic data for the 38 subjects was as follows: the mean onset age was 51.7±8.6 years (range 27-64 years) and there were 18 men and 20 women (male-female ratio 1:1.11). 28.9% of patients have a family history of dementia.

All patients were assessed by specialists in the field of dementia. Ten patients fulfilled the diagnostic criteria for probable AD [14], 9 patients fulfilled the diagnostic criteria for dementia but with uncertainty whether possible AD or FTD; the remaining 19 patients met the clinical criteria for the FTD disease spectrum: 11 for the behavior variant dementia (bvFTD) [15], one for semantic dementia (SD) [16], four for FTD combined with amyotrophic lateral sclerosis (FTD-ALS) [17] and three for FTD-parkinsonism overlap [18]. All available affected individuals were recruited from neurology clinics of Ruijin Hospital and Mental Health Center in Shanghai. The research was approved by the Ethics Committee of Ruijin Hospital and Mental Health Center, Shanghai Jiao Tong University in China. Written-informed consent was obtained from all subjects.

**Table 1:** Genes associated with dementia.

Phenotype	Genes
Early onset AD	APP, PSEN1, PSEN2
Late onset AD	APOE, A2M, ABCA7, ACE, APBB2, ATXN1, AKT1, AR, BIN1, BLMH, CASP3, CD2AP, CD33, CHCHD10, C9ORF72, CYP2C, CST3, CELF1, CLU, CR1, DNMT1, DSG2, EPHA1, ETS1, FERMT2, GSK3B, GRB2, HTR7, HFE, INPP5D, ITM2B, LRP1, MEF2C, MPO, MS4A4E, MPHOSPH1, MS4A6A, NME8, NOS3, NOTCH3, PICALM, PAXIP1, PLAU, PTK2B, SLC24A4, SORL1, TNF, TREM2, TYROBP, ZCWPW1
FTD	BTNL2, C9ORF72, CFS1R, CHCHD10, CHMP2B, CST3, CTSC, DCTN1, FUS, GRN, hnRNPA1, hnRNPA2B1, MAPT, OPTN, PRKAR1B, PRNP, RAB38, SIGMAR1, SOD1, SQSTM1, TBK1, TARDBP, TMEM106B, TREM2, UBQLN2, VCP
DLB	GBA, SNCA, SNCB
Other types	ATP13A2, EPM2A, ITM2B, NHLRC1, PRICKLE1, TRPM7

### Examinations

All patients received neuropsychological assessment: 24 patients received Mini-mental state examination (MMSE), seven of them received further tests including The Auditory Verbal Learning Test (AVLT), Taylor Complex Figure Test (CFT), Stroop Color Word Test (SCWT), Trail Making Test (TMT), Clock Drawing Task (CDT) and Boston Naming Test (BNT). The 14 patients with psychiatric symptoms received psychiatric rating scale evaluations including Wechsler Adult Intelligence Scale (WAIS) and Wechsler Memory Scale (WMS), Contingent Negative Variation (CNV), and Eysenck Personality Questionnaires (EPQ). Brain imaging and polymorphisms of apolipoprotein E (APOE) were analyzed in all patients. Blood tests (treponema pallidum hemagglutination assay, vitamin B_12_ levels, thyroid function and HIV) were conducted to exclude acquired causes of dementia.

**Figure 1.**
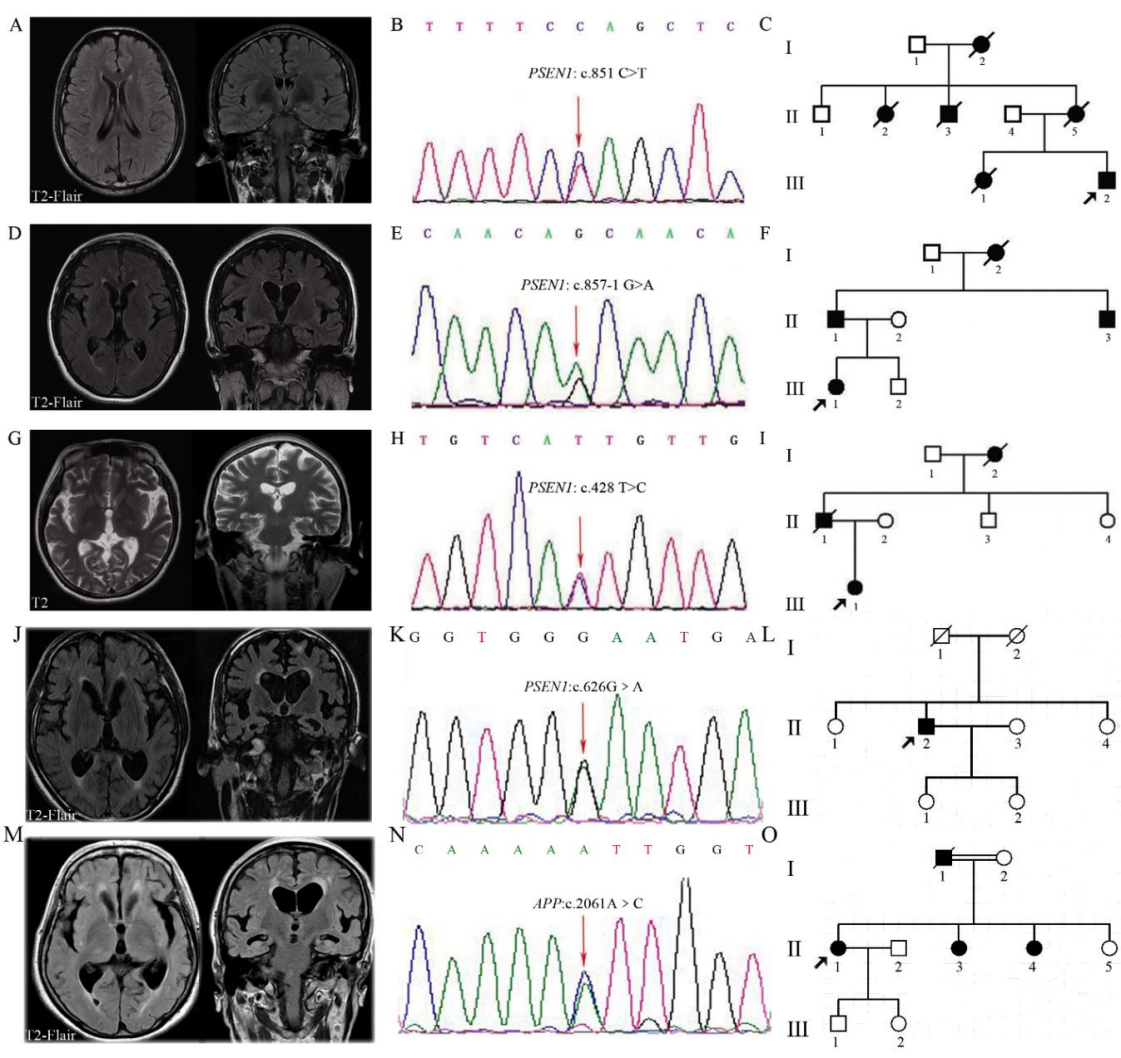
Magnetic resonance imaging (MRI), Alzheimer’s disease (AD) pathogenic mutations and Pedigrees for five cases Brain MRI (T2W-FLAIR) images from AT001 (**A**), AT002 (**D**), AT040 (**J**), AT045 (**M**) and brain MRI (T2WI) images from AT003 (G). (**B, E, H, K, N**) are the Sanger sequencing results of AT001, AT002, AT003, AT040, and AT045. (**C, F, I, L, O**) indicate pedigrees of AT001, AT002, AT003, AT040, and AT045. * T2W-FLAIR: T2 weighted fluid-attenuated inversion recovery; T2WI: T2-weighted imaging.

**Table 2:** Cases of variants with uncertain significance.

Case	Gene	Zygosity	Mutation (CDS)	Consequence at protein level	Clinical diagnosis	Frequency prediction			Software prediction			ACMG

						Esp6500	1000g2014	East Asian	Polyphen2	SIFT	Mutation taster	
AT008	*PSEN2*	Het	c.G640T	p.V214L	AD/FTD	NA	0.0012	0.002543	0.972/D	0.09/T	disease causing	VUS
AT017	*PSEN2*	Het	c.G640T	p.V214L	AD	NA	0.0012	0.002543	0.972/D	0.09/T	disease causing	VUS
AT029	*GRN*	Het	c.C1663T	p.R555W	SD	NA	NA	0.0002325	0.98/D	0.18/T	polymorphism	VUS
AT013	*TREM2*	Het	c.C331A	p.Q111K	AD	NA	NA	0.0002313	0.998/D	0.57/T	disease causing	VUS
AT015	*ABCA7*	Het	c.G5963T	p.C1988F	AD	NA	0.0009984	NA	1/D	0/D	disease causing	VUS
AT022	*TRPM7*	Het	c.C2525T	p.T842M	FTD-ALS	NA	NA	NA	0.945/P	0.08/T	disease causing	VUS
AT020	*NME8*	Het	c.1008dupT	p.R336fs	FTD-ALS	NA	NA	NA	NA	NA	disease causing	VUS
AT032	*SORL1*	Het	c.C3238T	p.R1080C	FTD	0.000077	NA	5.998e-05	0.992/D	0.06/T	disease causing	VUS
	*MPO*	Het	c.G980A	p.R327H		NA	NA	6.057e-05	1/D	0/D	disease causing	VUS
AT028	*APBB2*	Het	c.A433T	p.N145Y	FTD-parkinsonism	NA	NA	NA	0.561/P	0.03/D	disease causing	VUS
AT037	*ATP13A2*	Het	c.C2806T	p.T1483A	FTD	NA	NA	NA	1/D	0.18/T	disease causing	VUS
AT041	*PSEN2*	Het	c.C505A	p.H169N	AD	NA	0.00019968	0.002311	0.985/D	0.05/T	disease causing	VUS

Note. Polyohen2: probably damaging (D) [0.957,1]; possibly damaging (P) [0.453,0.956]; benign (B) [0, 0.452]; SIFT: damaging (D) [0 0.05); tolerated (T) [0.05-1]; CDS: coding sequence; Het: heterozygous; VUS: variant of uncertain significance; NA: not available; ESP: National Heart, Lung, and Blood Institute’s exome sequencing project; East Asian: the allelic frequencies of East Asian population in the Exome Aggregation Consortium database; 1000g=1000 Genomics Project; ACMG: the American College of Medical Genetics and Genomics standards and guidelines.

### Genetic analysis

To comprehensively investigate the potentially genetic cause of these patients, we summarized 89 dementia-related causing and susceptible genes using Online Mendelian Inheritance in Man (OMIM) and PubMed database (Table 1). DNA extraction from venous blood was performed using standard protocols. Repeated expansions in *C9ORF72* were detected adopting the methods as previously described [19]. An exon capture kit (Agilent SureSelect v5 reagents) was applied and captured libraries were sequenced on Illumina HiSeq X Ten platform. WES data were analyzed for single-nucleotide variants (SNVs) and insertion-deletions (InDels) in 89 dementia-related causing and susceptible genes. The significant results were comprehensively evaluated in aspects including minor allele frequency, conservation, predicted pathogenicity, disease association, confirmation with Sanger sequencing and familial segregation. These results were interpreted based on the ACMG standards and guidelines [20].

## RESULTS

In this research, we identified six pathogenic mutations and eleven variants with uncertain significance (Table 2). No pathogenic expansions in *C9ORF72* were detected. Among all our patients with dementia, 68.4% (26/38) patients developed an initial symptom of hypomnesia. 71.1% (27/38) patients developed behavioral changes and 28.9% (11/38) patients developed language defects during the disease.

### Phenotypes of AD patients and associated mutations

We identified four variants of uncertain significance in ten AD patients. AT017 with *PSEN2* (NM_000447: c.G640T, p.V214L) developed memory disturbance and apathy at age 52. AT013 became forgetful at age 47 and progressed to dementia at 51. He was identified to harbor *TREM2* (NM_001271821: c.C331A, p.Q111K). AT015 with *ABCA7* (NM_019112: c.G5963T, p.C1988F) and AT041 with *PSEN2* (NM_000447: c.C505A, p.H169N) was diagnosed AD at 59 and 64 respectively. Among the ten AD patients, five patients carried the APOE ε4/ε3 genotype; two patients carried APOE ε2/ε3; two patients carried the APOE ε4/ε4, and one patient carried APOE ε3/ε3 (Table 3).

**Table 3:** APOE genotypes of all patients.

Case	Diagnosis	Genetic result	APOE genotype
AT001	AD/FTD	*PSEN1*: NM_000021: exon8: c.C851T	ε3/ε3
AT002	AD/FTD	*PSEN1*: NM_007318: exon9: c.857-1G>A	ε3/ε3
AT003	AD/FTD	*PSEN1*: NM_000021: exon5: c.T428C	ε3/ε3
AT005	AD/FTD	None	ε4/ε3
AT006	AD/FTD	None	ε3/ε3
AT007	AD/FTD	None	ε3/ε3
AT008	AD/FTD	*PSEN2*: NM_000447: exon8: c.G640T	ε4/ε3
AT010	AD	None	ε4/ε3
AT011	AD	None	ε4/ε3
AT012	AD	None	ε4/ε3
AT013	AD	*TREM2*: NM_001271821: exon2: c.C331A	ε4/ε3
AT014	AD	None	ε2/ε3
AT015	AD	*ABCA7*: NM_019112: exon45: c.G5963T	ε4/ε3
AT017	AD	*PSEN2*: NM_000447: exon8: c.G640T	ε4/ε4
AT019	FTD-ALS	None	ε3/ε3
AT020	FTD-ALS	*NME8*: NM_016616: exon13: c.1008dupT	ε3/ε3
AT021	FTD-ALS	None	ε3/ε3
AT022	FTD-ALS	*TRPM7*: NM_017672: exon19: c.C2525T	ε4/ε3
AT025	FTD-CBS	None	ε3/ε3
AT026	FTD-PSPS	None	ε3/ε3
AT028	FTD-parkinsonism	*APBB2*: NM_001166051: exon6: c.A433T	ε3/ε3
AT029	SD	*GRN*: NM_002087: exon13: c.C1663T	ε2/ε3
AT030	bvFTD	None	ε3/ε3
AT031	bvFTD	None	ε3/ε3
AT032	bvFTD	*SORL1*: NM_003105: exon23: c.C3238T MPO: NM_000250: exon7: c.G980A	ε3/ε3
		*MPO*: NM_000250: exon7: G980A	
AT033	bvFTD	*MAPT*: NM_005910: exon13: G1165A NM_005910	ε2/ε3
AT034	bvFTD	None	ε3/ε3
AT035	bvFTD	None	ε3/ε3
AT036	bvFTD	None	ε3/ε3
AT037	bvFTD	*ATP13A2*: NM_001141974: exon25: c.C2806T	ε3/ε3
AT038	bvFTD	None	ε3/ε3
AT039	bvFTD	None	ε3/ε3
AT040	AD/FTD	*PSEN1*: NM_000021: exon7: c.G626A	ε4/ε3
AT041	AD	*PSEN2*: NM_000447: exon7: c.C505A	ε3/ε3
AT042	AD	None	ε4/ε4
AT043	bvFTD	None	ε3/ε3
AT044	AD	None	ε2/ε3
AT045	AD/FTD	*APP*: NM_000484: exon16: c.A2061C	ε2/ε3

### Phenotypes of FTD patients and associated mutations

We identified the *MAPT* pathogenic mutation in AT033 and verified the clinical diagnosis of FTD. We also detected seven variants of uncertain significance in *GRN, TRMP7, ATP13A2, SORL1, NME8, MPO* and *APBB2*. AT033 began to show behavioral deterioration at age 27. In the following months, he was found to be walking slowly and have language impairment. Physical examination revealed brisk reflexes, ankle clonus, positive left Babinski and incoordination in movements. His MMSE score was 17/30 with university culture. Brain MRI revealed mild atrophy of frontal and temporal lobes. There was no family history of similar diseases. Genetic testing revealed that the patient and his healthy father possessed the *MAPT* mutation (NM_005910: c.1165G>A, p.G389R).

AT029 developed naming difficulties, nonfluent language and visual hallucination in her sixties. MRI revealed predominant anterior temporal lobe atrophy. She was diagnosed with SD and was identified to harbor the variant *GRN* (NM_002087: c.C1663T, p.R555W). AT022 was admitted for her motor, cognitive, and behavioral deficits at age 63. She was clinically diagnosed with FTD-ALS after general assessment and was identified to carry a heterozygous variant in *TRPM7* (NM_017672: c.C2525T, p.T842M). Among the 19 FTD patients, 16 patients carried the APOE ε3/ε3 genotype; two patients carried APOE ε2/ε3 and one patient carried APOE ε4/ε3 (Table 3).

### Phenotypes of patients with uncertainty whether possible AD or FTD and associated mutations

We identified four *PSEN1* pathogenic mutations and one *APP* pathogenic mutation in five undiagnosed patients. We also detected one variant of uncertain significance in *PSEN2*. AT001 presented with short memory disturbance, acalculia, and nonfluent language at age 39. In the following two years, he suffered from slow response to external stimulations and bradykinesia in limbs. On examination, his speech was slow and nonfluent with excessive salvation. He also possessed positive Myerson’s sign and bilateral palm jaw reflections. His MMSE score was 16/30 with primary culture. Brain magnetic resonance imaging (MRI) revealed that multiple hyperintensity spots beside the bilateral lateral ventricles and in the subcortical area of frontal and parietal lobes. No lobar atrophy was observed (Fig.1A). Pittsburgh compound B (PiB) retention was negative. Several of his family members had developed bradykinesia, bedridden and died in their forties (Fig. 1C). The *PSEN1* mutation (NM_000021: c.851C?T, p.P284L) was found by genetic screening (Fig. 1B).

AT002 was admitted at age 41 with memory disturbance, acalculia, nonfluent language, slow response, and stiffness. In the previous 9 months, she was diagnosed with schizophrenia because of delusion of persecution and apatheia. During the therapy, she manifested generalized seizures. The neurological examination showed increased muscle tone of lower limbs, brisk tendon reflexes and positive Myerson’s sign. Rapid alternating movements of the fingers were slowed. Brain MRI showed moderate cerebral atrophy with a few subcortical white matter lesions (Fig. 1D). His father and uncle had also developed the similar symptoms (Fig. 1F). The *PSEN1* mutation (NM_007318: c.857-1G>A) was detected in the patient (Fig. 1E).

AT003 consulted the doctor at age 32 with two-year memory problems. She had difficulty remembering what happened in one or two days and even memories of her childhood. Her disposition became introverted and emotion was unstable. Involuntary tremors of right upper limbs appeared occasionally and progressed gradually. Recently, she developed visual hallucination. She scored 18 in MMSE and MRI revealed enlarged sulcus indicating generalized cerebral atrophy (Fig. 1G). A similar onset age and phenotype were described in her grandmother and father (Fig. 1I). The *PSEN1* mutation (NM_000021: c.428T?C, p.I143T) was identified in the patient (Fig. 1H).

AT040 developed hypomnesia at age 43. He had difficulties in understanding, calculation, and orientation progressively, and he developed nonfluent language and behavior changes in the following years. At age 47, he suffered from apatheia, bradykinesia, and gatism. He had increased muscle tone in his limbs, positive Myerson’s sign and positive right Babinski. Brain MRI showed global cerebral atrophy, obviously of the bilateral hippocampus, with multiple subcortical white matter hyperintensities in the temporal and parietal lobes and beside bilateral lateral ventricles (Fig. 1J). Fluorodeoxyglucose positron emission tomography (FDG-PET) showed decrease in glucose metabolism of the global cerebral, predominantly of the temporal, parietal and occipital lobes. The *PSEN1* mutation (NM_000021: c.626G?A, p.G209E) was identified in this patient (Fig. 1K).

AT045 developed short memory disturbance and naming difficulties at age 51. She could not figure out the time and position and could not recognize her relatives in one year. All her daily life required to be taken care of at age 53. Brain MRI revealed that global cerebral atrophy, obviously of the bilateral hippocampus, and white matter hyperintensities in subcortical cerebral and beside bilateral ventricles (Fig. 1M). Her father had the similar symptoms and died in his sixties. Her two sisters developed epilepsy and mental disturbance in their fifties respectively (Fig. 1O). The *APP* mutation (NM_000484: c.2061A?C, p.K687N) was identified in the patient and her sister (Fig. 1N).

AT008 with the variant of uncertain significance *PSEN2* (NM_000447: c.G640T, p.V214L) was referred to a neurologist at age 54 for deteriorating work performance. In the past 4 years, she became forgetful, apathetic and irritable. Her father, carrying the variant, did not show symptoms of dementia. Among these nine patients, five patients carried the APOE ε3/ε3 genotype; three patients carried APOE ε4/ε3 and one patient carried APOE ε2/ε3 (Table 3).

**Table 4:** Summary of basic characteristics of patients with V214L mutation in *PSEN2*.

Ethnicity	Sex	AO	Presenting symptoms	Family history	MMSE	APOE	Reference
Korean	F	69	Memory loss	NA	18	ε3/4	[39]
Korean	F	54	Memory loss, anomia	No	15	ε3/3	[37]
China	M	63	Memory loss	No	NA	ε3/3	[38]
China	F	64	Memory loss	Yes	NA	ε4/4	[38]
China	F	50	Memory loss and behavior changes	No	12	ε3/4	This study
China	M	48	Memory loss	No	15	ε4/4	This study

Note. F: female; M: male; AO: age at onset; NA: not available.

## DISCUSSION

Through WES in early-onset dementia patients, we genetically diagnosed five patients of atypical phenotypes of Alzheimer’s disease, including early onset ages, fast progressing, obvious psychiatric symptoms, prominent language deficiency or motor deficit during disease. Additionally, we verified the clinical diagnosis of the 27-year-old FTD patient and explored eleven variants of uncertain significance.

Amyloid precursor protein (*APP*), presenilin 1 (*PSEN1*), and presenilin 2 (*PSEN2*) mutations cause autosomal dominant EOAD. *PSEN1*-related EOAD with onset ages ranging 35-60 and accounts for 55% dominant EOAD cases [21]. *PSEN1* p.P284L and *PSEN1* c.857-1G>A mutations are reported to relate to Alzheimer’s disease with spastic paresis, which is pathologically characterized by large, noncored, weakly neuritic Aβ-amyloid plaques called “cotton-wool” amyloid plaques (CWP) [22-25]. *PSEN1* p.I143T is featured with particularly early dementia with a mean onset age of 34 years and the mean age of death of 41.2 years. The reported clinical characteristics include memory impairment, visuospatial disorientation, dyspraxia, dysphasia, as well as coordination problems including myoclonic jerks and multiple falls. The pathological feature of this mutation is the high production ratio of Aβ 42/40 and increased levels of Aβ 43 in the examined brain area [23, 26]. The phenotype of our patient with *PSEN1* p.G209E was characterized with frontal symptoms such as personality change, temporal symptoms such as nonfluent languages, parietal symptoms such as agnosia and the presentation of extrapyramidal syndrome, which was more serious than the reported phenotype caused by G209R and G209V mutations in*PSEN1* [27].

*PSEN1* mutation carriers are prone to present with atypical cognitive symptoms of behavioral change, language impairment, dyscalculia or executive impairment, and other neurological features of myoclonus, seizures, and pyramidal, extrapyramidal or cerebellar signs. Extremely early onset ages are observed for mutations before codon 200 in *PSEN1* and atypical presentations of dementia for mutations beyond codon 200 [28, 29]. Both AT001 and AT002 developed bradykinesia and nonfluent language in their forties. AT002’s disease progression was more aggressive for she manifested personality changes, spastic gait at the early stage of the disease. The functional experiment demonstrated that *PSEN1* c.857-1G>A caused genomic deletion of presenilin 1 exon 9, which led to the serious form of dementia [30]. Notably, the reported Japanese patient with *PSEN1* P284L who developed spastic paresis preceded dementia at her thirties [24]. Comparably, AT001 with the same mutation did not show symptoms of spasticity though he had developed amnestic symptoms for two years. Our observations of four families with *PSEN1* mutations reveal the highly variable clinical phenotypes with *PSEN1* mutations.

*APP* (c.2061A?C, p.K687N) was a novel mutation detected. AT045 carrying the mutation developed memory disturbance at age 51 and progressed so fast that she could not recognize her families and lost self-living abilities in two years. AT045’s sister with the mutation developed seizures without inducing factors, while her cognitive competence was mildly impaired at age 53. The siblings carrying *APP* mutation displayed different initial clinical manifestations. Seizures are a common feature of EOAD yet no patient for *APP* missense mutation has been reported [31]. Our investigations broaden the phenotype of AD patients with *APP* mutations.

Another important observation was that subcortical white matter hyperintensities (WMH) existed in the brain imaging of AT001, AT002, AT040, and AT045, but was absent in AT003. WMH have been pathologically linked to ischemia, infarction, demyelination, and edema. It can be a core feature of Alzheimer’s disease and share some degree of dependency with amyloid-β (Aβ) pathology [32]. Widespread white matter abnormalities have been shown to associate with *PSEN1* mutation-related spastic paraparesis and the degree of WMH has positive correlations with the severity of CWP [33, 34]. Previous pathological studies reported a particularly severe cerebral amyloid angiopathy (CAA) with post codon 200 *PSEN1* mutations and amyloid beta coding domain *APP* mutations. CAA may manifest as WMH on magnetic resonance imaging [35]. All these conclusions indicate the underlying pathological heterogeneity in different mutation sites.

Importantly, we explored eleven mutations to be classified as variants of uncertain significance, which may contribute to the disease occurrence. Unlike *PSEN1* carriers, mutations in *PSEN2* cause AD with variable penetrance and have a later onset age [36]. Two Chinese patients and one Korean patient with *PSEN2* p.V214L have been identified in the Chinese AD cohort consisting of 61 patients and Korean EOAD cohort consisting of 104 patients respectively, indicating that *PSEN2* p.V214L could have associations with the risks of developing AD in the Asian population. Moreover, structural prediction of *PSEN2* V214L revealed important structural changes affecting adjacent amino acids [37-39]. Including the reported patients and our patients with *PSEN2* V214L, the onset ages among these patients ranged from 48 years to 69 (Table 4). Notably, AT008’s father carrying the mutation and ε4/ε3 genotype did not develop dementia. Thus, it is speculated that other important modifying factors may contribute to disease penetrance and progression.

It has been reported that pathogenic missense mutations in *GRN* possess the milder effect on GRN structure or function compared to a loss of functional mutations [40]. The Arg555 residue was in GRN-domains and the mutation may affect protein function based on Polyphen2 and SIFT analysis. We did not detect the *GRN* p.R555W variant in 200 healthy controls with the same ethnic background. Therefore, in the absence of functional data, we cannot conclude that this patient-specific missense mutation merely presents rare polymorphism.

TRPM7 channels are selective to Ca^2+^ and Mg^2+^, and the discrepancy in TRPM7 channel function and expression leads to various neuronal diseases such as AD, Parkinson’s disease (PD) and ALS [41]. We explored a heterozygous mutation in *TRPM7* (c.2525C>T, p.T842M) in an FTD-ALS patient. The variant was predicted to be detrimental by PolyPhen2 and Mutation Taster and was not identified in 200 healthy controls. Further studies are required to clarify the mechanisms involved and the associations between *TRPM7* and FTD.

Using clinical and genetic approaches, our findings indicate that mutations in *PSEN1* and *APP* can lead to atypical clinical symptoms at an early stage and a serious form of dementia. However, we did not explore the potential genetic cause in 84.2% patients with early-onset dementia. Therefore, several factors should be considered. First, the pathogenicity of variants with uncertain significance should be verified by large-scale studies and fundamental experiments. Second, the limitations of next-generation sequencing technology for large segment insertion or deletion detection may fail to explore the genetic cause in a subset of these patients [42]. Thirdly, a subset of probands might have been explained by novel dementia genes that are yet to be identified. The secondary analysis of WES data in time is necessary for enhancing positive molecular diagnosis rate.

In conclusion, next-generation techniques have the potential to give a genetic diagnosis for some intractable clinical cases caused by the heterogeneity of clinical manifestations and incomplete penetrance of genetics. The current study also expands the clinical spectrum of EOAD and FTD.
